# Real-world evidence in gynecologic cancers presented at key oncology conferences in the United States: Distribution and factors related to high-tier acceptance

**DOI:** 10.1371/journal.pone.0321654

**Published:** 2025-04-22

**Authors:** Elizabeth A. Szamreta, Mansi Modi, Ramu Periyasamy, Bhavani Yamsani, Pattabhi Machiraju, Neetu Menghani, Matthew Monberg

**Affiliations:** 1 Outcomes Research, Merck & Co., Inc., Rahway, New Jersey, United States of America; 2 Ernest Mario School of Pharmacy, Rutgers University, Piscataway, New Jersey, United States of America; 3 Medical Affairs, Indegene Ltd, Bangalore, Karnataka, India; Chunghwa Telecom Co. Ltd., TAIWAN

## Abstract

**Purpose:**

To describe the distribution, trends, and characteristics of types of real-world evidence (RWE) abstracts presented at key oncology congresses.

**Methods:**

Data on gynecologic cancers (cervical, ovarian, endometrial, and multiple gynecologic/other) were extracted from the American Society of Clinical Oncology (ASCO) and the Society of Gynecologic Oncology (SGO) conference databases (2018–2020) to: a) identify the proportion of clinical trial (CT) versus RWE abstracts accepted; b) describe the distribution and tier of acceptance of RWE versus CTs; c) analyze the characteristics (authorship, data source, data type, study design, outcome[s], and presence of statistically significant results) associated with RWE acceptance.

**Results:**

Of 3163 abstracts screened, 2271 (77% RWE, 23% CTs) were included. RWE represented a higher proportion of work at SGO versus ASCO (70% vs 30%). Overall, more RWE studies versus CTs were accepted as posters (75% vs 60%), while fewer were accepted as oral presentations (4% vs 20%; *p* < 0.001 for both). Among RWE abstracts, 90% had academic author(s), 68% of studies were from North America, 45% used other clinical data sources, and nearly 32% reported statistically significant results. Approximately 60% of RWE were retrospective and 9% were prospective. The most common outcomes in RWE abstracts were molecular analyses (18%) and survival based on treatment efficacy (13%; *p* < 0.001).

**Conclusion:**

RWE abstracts were accepted for presentation more frequently at SGO versus ASCO, and majority of them were presented as posters. While RWE abstracts are prevalent and provide valuable data for healthcare decision-making, they do not always achieve the visibility of CTs.

## Introduction

Real-world data (RWD) has garnered growing interest in the oncology community in addressing clinical and policy-relevant queries that cannot be resolved with clinical trial (CT) data [1]. Although randomized controlled trials (RCTs) are the accepted gold standard for generating clinical evidence [2], the controlled research settings, resource intensiveness, and limited follow-up duration restrict their generalizability to real-world clinical practice [2–4]. In contrast, real-world evidence (RWE) provides insights in a short time with lesser resources and reduced costs compared to RCTs [2].

Recently, there have been significant investments in electronic health record (EHR) capabilities for oncology data collection, leading to databases with very large sample sizes [5]. Furthermore, RWD sources such as EHRs of treatment and outcomes linked to cancer registry data provide insights into patients, treatments, and related outcomes in routine oncology practice [1]. Real-world issues addressed by RWE studies in diverse geographic and/or economic contexts include cancer burden, access to cancer care, quality of care and/or adherence, long-term safety, efficacy, and cost-effectiveness [1,3,4]. Additionally, RWE studies can describe treatment efficacy and tolerability in special patient populations which are either under-represented or excluded from RCTs [4].

While RWE is becoming increasingly important in healthcare decision-making, its value is still not always recognized. RWE studies complement RCTs by providing insights with a high degree of external validity as well as for a better understanding of the biological effects of a drug in different settings [2,6]. Despite the increasing presence of RWE in oncology, there remains a paucity of studies comparing the RWE landscape with CTs and evaluating the factors associated with their high-tier acceptance at scientific conferences, particularly in gynecologic cancers. Analyzing the type of research accepted, the number of abstracts, their trends and characteristics, and the tier of presentations at key oncology conferences can help understand the priority of these scientific presentations, thus allowing for better dissemination of different types of research.

Prior studies have focused on assessing the publication patterns of oncology and/or hematology abstracts presented at major conferences to identify the factors that influence their publication rates [7–11]. Previous meta-research by Frank et al., 2022 evaluated the association of accuracy estimates, abstract conclusion positivity, and completeness of abstract reporting with acceptance at radiology conferences [12]. A careful evaluation of the trends of abstract presentation is critical as they have the potential to influence clinical decision-making and research [13]. Hence, the primary objective of this study is to evaluate the proportion of RWE versus CT abstracts presented at two annual oncology conferences, the American Society of Clinical Oncology (ASCO) and the Society of Gynecologic Oncology (SGO). The secondary objectives are to describe the distribution and trends of RWE abstracts and the tier of acceptance for RWE versus CT and to analyze the association between RWE abstract characteristics and the different tiers of acceptance.

## Methods

### Study design

A literature review was conducted in November 2021 to identify the abstracts accepted at ASCO and SGO annual medical conferences between 2018 and 2020, to assess the type of data within each abstract and the format of presentation at the conference. To narrow our focus, we selected specific gynecologic cancers: ovarian, cervical, and endometrial cancers as a case study, and the abstracts focusing on these cancers were included. Therefore, the SGO conference (a prominent annual meeting on gynecologic cancer in the United States [US]) was considered for this study as it primarily focuses on gynecologic cancers, and ASCO, being a broader oncology conference, was selected as a comparator. The selected time period was chosen to depict the recent trends, including those in the coronavirus disease (COVID)-19 era virtual conferences as well as pre-COVID times. Since this study was based on a literature review of publicly available abstracts from the ASCO and SGO annual medical conferences (2018–2020), and no human participants or personal data were involved, institutional review board approval and participant consent were not required.

### Data source and search strategy

A systematic literature search was performed using Embase, SGO, and ASCO databases to identify relevant abstracts. The search strategy included multiple keywords and combinations thereof: cervical, ovarian, endometrial, and cancer/tumor/tumour/carcinoma (S1 Table).

### Eligibility criteria

The inclusion criteria were based on the patient, intervention, comparison, outcome, time, and study design (PICOTS) framework. Abstracts including preclinical data, *in vitro*/*ex vivo*/*in silico* experiments, non-gynecological cancer types, and gender-diverse populations were excluded. Terms related to CTs and RWE (S1 Table) were used to classify the abstracts as CT or RWE. Studies either on multiple gynecologic cancers of interest (ovarian, cervical, and endometrial) or those including a combination of these and other types of cancer were included under the multiple cancers category.

### Study selection

The search results retrieved from the data sources were collated using MS Office Excel for deduplication. The unique records were then screened for eligibility based on titles and content by two independent reviewers. Any disagreements between the reviewers were resolved through discussion until a consensus was reached.

### Data extraction

Both clinical and RWE abstracts were included from the ASCO and SGO conference databases to determine the proportion of each type among accepted studies. Data (year of publication, cancer type, and tier of acceptance) from the selected studies were extracted by two independent reviewers into standardized MS Office Excel for both CT and RWE abstracts after reaching a consensus on the eligibility of the study. The broad categories for the tier of acceptance were defined a priori and included education forum, international session, abstract (late-breaking abstract [LBA] session, LBA, abstract), oral (LBA-sunrise session, LBA-scientific plenary, clinical science symposium, focused plenary, focused plenary session, oral abstract session, seminal abstracts, scientific plenary, sunrise session), poster discussion session, poster session (poster session, featured poster session, poster), publication only and others category (special interest session, and sunrise seminar). A LBA is an abstract presenting new information not available/fully known by the original abstract submission deadline. The education forum, international session, and “others” category were from SGO only, and the poster discussion session was specific to ASCO.

Additional characteristics collected for RWE studies only included: single- or multi-site study, academic authorship, country(ies) of study conduct and data source, industry sponsorship, type of data collected (chart review, medical records, patient registry, claims data, billing data, EHR, electronic medical records, interview, survey, literature, questionnaires, other data repository or primary data collection, and other clinical data sources), the presence of statistically significant results, type of analysis/RWE study design, sample size, and endpoint(s) or outcome(s) assessed. “Other clinical data sources” included abstracts involving the collection of human biological specimens (such as blood, plasma, serum, urine, tissues [including paraffin blocks], DNA/RNA, or any other cells or fluids). “Other data repository” included abstracts reporting data collection from national or institutional databases (such as comprehensive cancer-related, surveillance, epidemiological, or genomic profiling).

The type of analysis/study design included comparative effectiveness, diagnosis, disease burden and epidemiology, economic model, prospective or retrospective, patient-reported outcomes (PROs), survival, systematic review, meta-analysis, treatment patterns, and others (categories different from earlier defined types, e.g., discrete choice experiment).

### Statistical analysis

Descriptive statistics were reported for the study variables using frequencies and percentages. Fisher’s exact or Chi-square test of independence was used to examine the statistically significant differences (*p* < 0.05, two-tailed) between the two groups (RWE vs. CT). All statistical analyses were performed using R 4.2.1 software.

## Results

### Distribution and trends of RWE and CT abstracts

A total of 3163 abstracts were screened, of which 2271 were included in the analysis (RWE abstracts, n = 1756 [77.32%]; CTs, n = 515 [22.68%]). The proportion of RWE abstracts was higher at SGO compared with ASCO (69.99% vs 30.01%), while CT abstracts represented a higher proportion of abstracts from ASCO compared with SGO (72.43% vs 27.57%; *p* < 0.001 for both). Overall, there was an increasing trend of RWE abstract acceptance from 2018 to 2020 (2018 vs 2019 vs 2020: 27.16% vs 33.6% vs 39.24%; *p* < 0.001) (Table 1). Of abstracts accepted overall, the number of total abstracts increased by 10.52%, the proportion of RWEs by 9.34%, and the proportion of CTs by 1.19% from 2018 to 2020. In general, abstracts on ovarian cancer represented a higher proportion of those accepted than abstracts on other gynecologic cancer types (*p* < 0.001). Additionally, the proportion of RWE abstracts on ovarian cancer was higher compared with other cancer types; whereas among CTs, the highest were on multiple cancers versus all the gynecological cancer types studied (*p* < 0.001 for both) (Table 1).

**Table 1:** Distribution of abstracts stratified by CT and RWE, conference, year of publication, cancer type, and tier of acceptance.

2019	Variables, n (%)	Total (N = 2271) n (%)	CTs (n = 515) n (%)	RWE (n = 1756) n (%)	Statistical test	Test statistic	*p*-value
**Conference**	ASCO	900 (39.63)	373 (72.43)	527 (30.01)	Chi-square	*χ*^2^ (1) = 297.680	< 0.001
	SGO	1371 (60.37)	142 (27.57)	1229 (69.99)			
**Year**	2020	886 (39.01)	197 (38.25)	689 (39.24)		*χ*^2^ (2) = 7.771	< 0.05
	2019	738 (32.5)	148 (28.74)	590 (33.6)			
	2018	647 (28.49)	170 (33.01)	477 (27.16)			
**Cancer type**	Cervical cancer	382 (16.82)	57 (11.07)	325 (18.51)		*χ*^2^ (3) = 69.865	< 0.001
	Ovarian cancer	834 (36.72)	196 (38.06)	638 (36.33)			
	Endometrial cancer	422 (18.58)	56 (10.87)	366 (20.84)			
	Multiple cancers	633 (27.87)	206 (40.0)	427 (24.32)			
**Tier of acceptance**	Oral	170 (7.49)	101 (19.61)	69 (3.93)	Fisher’s exact	NA	< 0.001
	Poster Discussion Session	52 (2.29)	42 (8.16)	10 (0.57)			
	Poster Session	1622 (71.42)	307 (59.61)	1315 (74.89)			
	International Session	17 (0.75)	2 (0.39)	15 (0.85)			
	Education Forum	16 (0.7)	6 (1.17)	10 (0.57)			
	Publication only/Abstract	385 (16.95)	57 (11.07)	328 (18.68)			
	Others^a^	9 (0.4)	0	9 (0.51)			

Abbreviations: ASCO, American Society of Clinical Oncology; CTs, clinical trials; NA, not applicable; RWE, real-world evidence; SGO, Society of Gynecologic Oncology.

^a^Special interest session and Sunrise seminar were combined into the “Others category”.

Overall, the majority of the abstracts (71.42%) were accepted as poster sessions compared with other tiers of acceptance (*p* < 0.001). A greater proportion of RWE abstracts compared with CTs were accepted as posters (74.89% vs 59.61%) or publication only/abstract (18.68% vs 11.07%), and fewer as oral presentations (3.93% vs 19.61%; *p* < 0.001 for all) (Table 1).

Further, at ASCO, the majority of the RWE abstracts were accepted as publication only/abstract compared with CTs; CTs were accepted more often as poster sessions and oral presentations than RWE across various cancer types assessed (*p* < 0.001 for all) (Table 2). However, at SGO, a higher proportion of RWE abstracts were accepted as poster sessions compared to CTs (Table 2).

**Table 2:** Proportion of CTs versus RWE abstracts accepted at ASCO and SGO by year of publication, cancer type, and tier of acceptance.

	Acceptance at ASCO						Acceptance at SGO					
**Total** **(N = 900)** **n (%)**	**CTs** **(n = 373)** **n (%)**	**RWE** **(n = 527)** **n (%)**	**Statistical test**	**Test statistic**	***p*-value**	**Total** **(N = 1371)** **n (%)**	**CTs** **(n = 142)** **n (%)**	**RWE** **(n = 1229)** **n (%)**	**Statistical test**	**Test statistic**	***p*-value**
**Year**												
2020	307 (34.11)	137 (36.73)	170 (32.26)	Chi-square	*χ*^2^ (2) = 11.76	< 0.005	579 (42.23)	60 (42.25)	519 (42.22)	Chi-square	*χ*^2^ (2) = 0.0801	0.9607
2019	311 (34.6)	105 (28.15)	206 (39.09)				427 (31.15)	43 (30.28)	384 (31.25)			
2018	282 (31.33)	131 (35.12)	151 28.65)				365 (26.62)	39 (27.46)	326 (26.53)			
**Cancer type and tier of acceptance**												
**Cervical cancer**	**119 (13.22)**	**35 (9.38)**	**84 (15.94)**	Fisher’s exact	NA	< 0.001	**263 (19.18)**	**22 (15.49)**	**241 (19.61)**	Fisher’s exact	NA	< 0.001
Oral	60 (50.42)	2 (5.71)	3 (3.57)				14 (5.32)	7 (31.82)	7 (2.9)			
Poster Discussion Session	50 (42.02)	3 (8.57)	1 (1.19)				NA	NA	NA			
Poster Session	5 (4.2)	24 (68.57)	26 (30.95)				235 (89.35)	13 (59.09)	222 (92.12)			
International Session	NA	NA	NA				8 (3.04)	1 (4.55)	7 (2.9)			
Education Forum	NA	NA	NA				1 (0.38)	1 (4.55)	0			
Publication only/Abstract	4 (3.36)	6 (17.14)	54 (64.29)				0	0	0			
Others^a^	NA	NA	NA				5 (1.9)	0	5 (2.07)			
**Ovarian cancer**	**308 (34.22)**	**129 (34.58)**	**179 (33.97)**	Fisher’s exact	NA	< 0.001	**526 (38.37)**	**67 (47.18)**	**459 (37.35)**	Fisher’s exact	NA	< 0.001
Oral	21 (6.82)	18 (13.95)	3 (1.68)				47 (8.94)	23 (34.33)	24 (5.23)			
Poster Discussion Session	26 (8.44)	23 (17.83)	3 (1.68)				NA	NA	NA			
Poster Session	143 (46.43)	69 (53.49)	74 (41.34)				451 (85.74)	37 (55.22)	414 (90.2)			
International Session	NA	NA	NA				5 (0.95)	0	5 (1.09)			
Education Forum	NA	NA	NA				9 (1.71)	1 (1.49)	8 (1.74)			
Publication only/Abstract	118 (38.31)	19 (14.73)	99 (55.31)				12 (2.28)	6 (8.96)	6 (1.31)			
Others^a^	NA	NA	NA				2 (0.38)	0	2 (0.44)			
**Endometrial cancer**	**82 (9.11)**	**30 (8.04)**	**52 (9.87)**	Fisher’s exact	NA	< 0.001	**340 (24.80)**	**26 (18.31)**	**314 (25.55)**	Fisher’s exact	NA	< 0.001
Oral	6 (7.32)	6 (20)	0				26 (7.65)	12 (46.15)	14 (4.46)			
Poster Discussion Session	2 (2.44)	1 (3.33)	1 (1.92)				NA	NA	NA			
Poster Session	41 (50)	23 (76.67)	18 (34.62)				307 (90.29)	14 (53.85)	293 (93.31)			
International Session	NA	NA	NA				1 (0.29)	0	1 (0.32)			
Education Forum	NA	NA	NA				2 (0.59)	0	2 (0.64)			
Publication only/Abstract	33 (40.24)	0	33 (63.46)				3 (0.88)	0	3 (0.96)			
Others^a^	NA	NA	NA				1 (0.29)	0	1 (0.32)			
**Multiple cancers**	**391 (43.44)**	**179 (47.99)**	**212 (40.23)**	Fisher’s exact	NA	< 0.001	**242 (17.65)**	**27 (19.01)**	**215 (17.49)**	Fisher’s exact	NA	< 0.001
Oral	26 (6.65)	22 (12.29)	4 (1.89)				25 (10.33)	11 (40.74)	14 (6.51)			
Poster Discussion Session	20 (5.12)	15 (8.38)	5 (2.36)				NA	NA	NA			
Poster Session	189 (48.34)	118 (65.92)	71 (33.49)				206 (85.12)	9 (33.33)	197 (91.63)			
International Session	NA	NA	NA				3 (1.24)	1 (3.7)	2 (0.93)			
Education Forum	NA	NA	NA				4 (1.65)	4 (14.81)	0			
Publication only/Abstract	156 (39.9)	24 (13.41)	132 (62.26)				3 (1.24)	2 (7.41)	1 (0.47)			
Others^a^	NA	NA	NA				1 (0.41)	0	1 (0.47)			

Abbreviations: ASCO, American Society of Clinical Oncology; CTs, clinical trials; NA, not applicable; RWE, real-world evidence; SGO, Society of Gynecologic Oncology.

^a^Special interest session and Sunrise seminar were combined into the “Others category”.

Among the RWE studies, 53.47% were conducted at single sites, 90.03% had academic authorship, and two-thirds (67.65%) of the data sources of the abstracts were from North America (S1 Fig).

### Characteristics of RWE abstracts

#### Data types.

Overall, data from other clinical data sources were most frequently utilized in RWE abstracts (44.59%), followed by other data repositories (21.53%) and medical records (6.66%;

*p* < 0.001 for all; Table 3). About three-quarters of the RWE studies that included data from other clinical data sources were presented as poster sessions (74.58%), followed by publication only/abstracts (19.03%). Overall, there was no statistically significant association between the type of data used and the tier of presentation (*p* = 0.25) (S2 Table).

**Table 3:** Distribution of RWE abstracts by type of data collected and cancer type.

Type of data collected	Total (N = 1756) n (%)	Cervical cancer (n = 325) n (%)	Ovarian cancer (n = 638) n (%)	Endometrial cancer (n = 366) n (%)	Multiple cancers (n = 427) n (%)	*p*-value
Other clinical data sources	783 (44.59)	152 (46.77)	279 (43.73)	161 (43.99)	191 (44.73)	< 0.001
Other data repository	378 (21.53)	73 (22.46)	139 (21.79)	95 (25.96)	71 (16.63)	
Medical records	117 (6.66)	30 (9.23)	48 (7.52)	14 (3.83)	25 (5.85)	
Chart review	105 (5.98)	14 (4.31)	26 (4.08)	28 (7.65)	37 (8.67)	
Other primary data collection	88 (5.01)	14 (4.31)	41 (6.43)	10 (2.73)	23 (5.39)	
Survey	82 (4.67)	9 (2.77)	23 (3.61)	11 (3.01)	39 (9.13)	
Patient registry	53 (3.02)	14 (4.31)	16 (2.51)	14 (3.83)	9 (2.11)	
Others^a^	47 (2.68)	10 (3.08)	20 (3.13)	8 (2.19)	9 (2.11)	
EMR	31 (1.77)	4 (1.23)	15 (2.35)	7 (1.91)	5 (1.17)	
Claims data	20 (1.14)	0	12 (1.88)	3 (0.82)	5 (1.17)	
Interview	17 (0.97)	3 (0.92)	6 (0.94)	3 (0.82)	5 (1.17)	
Medical records and patient registry	9 (0.51)	0	3 (0.47)	5 (1.37)	1 (0.23)	
Literature	9 (0.51)	2 (0.62)	4 (0.63)	1 (0.27)	2 (0.47)	
Billing data	6 (0.34)	0	1 (0.16)	4 (1.09)	1 (0.23)	
Questionnaire	6 (0.34)	0	1 (0.16)	2 (0.55)	3 (0.7)	
EHR	5 (0.28)	0	4 (0.63)	0	1 (0.23)	

Abbreviations: EHR, electronic health record; EMR, electronic medical record; RWE, real-world evidence.

Fisher’s exact test was applied to assess the association between the distribution of RWE abstracts based on the type of data collected and cancer type.

^a^Others category included abstracts that could not be classified into the various categories of “Type of data collected”.

#### Statistical significance of results.

Of the RWE abstracts analyzed, nearly one-third reported statistically significant results while only a few contained no statistically significant results (32.35% vs 6.38%; *p* < 0.001). Furthermore, 31.49% of RWE abstracts did not report a statistical analysis of results, and the proportion of these RWE studies was higher at ASCO compared with SGO (41.18% vs 27.34%; *p* < 0.001) (Table 4). Most of the RWE abstracts were accepted as poster sessions regardless of the statistical significance (*p* < 0.001 for all) (Table 4). However, in SGO, the majority of the abstracts that reported statistically significant results (92.7%) were accepted as poster sessions versus other tiers of acceptance, whereas in ASCO, more than half (59.06%) of the RWE abstracts with statistically significant results were accepted as publication only/abstract (*p* < 0.001 for both) (Table 4).

**Table 4:** Distribution of RWE abstracts by conference, statistical significance of results, and tier of acceptance.

Were results statistically significant?	Total (N = 1756) n (%)	ASCO (n = 527) n (%)	SGO (n = 1229) n (%)	*p*-value
**Yes**	**568 (32.35)**	**171 (32.45)**	**397 (32.3)**	< 0.001
Oral	20 (3.52)	3 (1.75)	17 (4.28)	
Poster Discussion Session	2 (0.35)	2 (1.17)	NA	
Poster Session	433 (76.23)	65 (38.01)	368 (92.7)	
International Session	1 (0.18)	NA	1 (0.25)	
Education Forum	3 (0.53)	NA	3 (0.76)	
Publication only/Abstract	107 (18.84)	101 (59.06)	6 (1.51)	
Others^a^	2 (0.35)	NA	2 (0.5)	
**No**	**112 (6.38)**	**26 (4.93)**	**86 (7.0)**	< 0.001
Oral	1 (0.89)	0	1 (1.16)	
Poster Discussion Session	0	0	NA	
Poster Session	94 (83.93)	10 (38.46)	84 (97.67)	
International Session	1 (0.89)	NA	1 (1.16)	
Education Forum	0	NA	0	
Publication only/Abstract	16 (14.29)	16 (61.54)	0	
Others^a^	0	NA	0	
**Both**	**523 (29.78)**	**113 (21.44)**	**410 (33.36)**	< 0.001
Oral	25 (4.78)	5 (4.42)	20 (4.88)	
Poster Discussion Session	1 (0.19)	1 (0.88)	NA	
Poster Session	417 (79.73)	40 (35.4)	377 (91.95)	
International Session	5 (0.96)	NA	5 (1.22)	
Education Forum	3 (0.57)	NA	3 (0.73)	
Publication only/Abstract	70 (13.38)	67 (59.29)	3 (0.73)	
Others^a^	2 (0.38)	NA	2 (0.49)	
**Not available**	**553 (31.49)**	**217 (41.18)**	**336 (27.34)**	< 0.001
Oral	23 (4.16)	2 (0.92)	21 (6.25)	
Poster Discussion Session	7 (1.27)	7 (3.23)	NA	
Poster Session	371 (67.09)	74 (34.1)	297 (88.39)	
International Session	8 (1.45)	NA	8 (2.38)	
Education Forum	4 (0.72)	NA	4 (1.19)	
Publication only/Abstract	135 (24.41)	134 (61.75)	1 (0.3)	
Others^a^	5 (0.9)	NA	5 (1.49)	

Abbreviations: ASCO, American Society of Clinical Oncology; NA, not applicable; RWE, real-world evidence; SGO, Society of Gynecologic Oncology.

Fisher’s exact test was applied to assess the association between the distribution of RWE abstracts based on the conference, statistical significance of results, and tier of acceptance.

^a^Special interest session and Sunrise seminar were combined into the “Others category”.

#### Type of analysis.

In general, RWE abstracts with a retrospective study design or analyses represented a greater proportion of the accepted RWE abstracts than the prospective study design (59.97% vs 9.4%). Retrospective RWEs were more represented at SGO than ASCO (64.36% vs 49.72%); while prospective abstracts represented similar proportions of accepted work at both conferences. Overall, RWE abstracts related to diagnosis (16.0%) represented a high proportion of accepted work, followed by PROs (4.10%), and economic models (1.65%) (Table 5). Among retrospective RWEs (n = 1053), the majority of the abstracts (78.25%) were presented as poster sessions followed by publication only/abstract (16.52%) and orals (3.13%; *p* < 0.01). In contrast, RWE abstracts on economic models (n = 29) were represented more as orals than publication only/abstract (17.24% vs 10.34%; *p* < 0.01) (S3 Table) and were presented more at SGO than ASCO (n = 23 [1.87%] vs n = 6 [1.14%]) (Table 5). Furthermore, 80.7% of RWE abstracts on survival were presented as posters and only 3.51% as orals (S3 Table).

**Table 5:** Distribution of RWE abstracts by conference, type of analysis, and cancer type.

	Total N (%)	Cervical cancer n (%)	Ovarian cancer n (%)	Endometrial cancer n (%)	Multiple cancers n (%)	*p*-value
**ASCO**	**527 (30.01)**	**84 (15.94)**	**179 (33.97)**	**52 (9.87)**	**212 (40.23)**	< 0.01
Retrospective	262 (49.72)	47 (55.95)	92 (51.4)	33 (63.46)	90 (42.45)	
Diagnosis	113 (21.44)	13 (15.48)	26 (14.53)	7 (13.46)	67 (31.6)	
Prospective	49 (9.3)	7 (8.33)	19 (10.61)	3 (5.77)	20 (9.43)	
PROs	31 (5.88)	7 (8.33)	7 (3.91)	1 (1.92)	16 (7.55)	
Survival	20 (3.8)	4 (4.76)	9 (5.03)	1 (1.92)	6 (2.83)	
Meta-analysis	12 (2.28)	0	7 (3.91)	1 (1.92)	4 (1.89)	
Comparative effectiveness	10 (1.9)	1 (1.19)	5 (2.79)	3 (5.77)	1 (0.47)	
Treatment patterns	9 (1.71)	3 (3.57)	2 (1.12)	2 (3.85)	2 (0.94)	
Economic model	6 (1.14)	1 (1.19)	4 (2.23)	0	1 (0.47)	
Literature review	6 (1.14)	0	2 (1.12)	1 (1.92)	3 (1.42)	
Others	4 (0.76)	0	3 (1.68)	0	1 (0.47)	
Disease epidemiology	3 (0.57)	1 (1.19)	2 (1.12)	0	0	
Systematic review	2 (0.38)	0	1 (0.56)	0	1 (0.47)	
**SGO**	**1229 (69.99)**	**241 (19.61)**	**459 (37.35)**	**314 (25.55)**	**215 (17.49)**	< 0.001
Retrospective	791 (64.36)	172 (71.37)	283 (61.66)	208 (66.24)	128 (59.53)	
Diagnosis	168 (13.67)	23 (9.54)	67 (14.6)	54 (17.2)	24 (11.16)	
Prospective	116 (9.44)	19 (7.88)	38 (8.28)	25 (7.96)	34 (15.81)	
PROs	41 (3.34)	4 (1.66)	15 (3.27)	8 (2.55)	14 (6.51)	
Survival	37 (3.01)	6 (2.49)	18 (3.92)	8 (2.55)	5 (2.33)	
Economic model	23 (1.87)	4 (1.66)	14 (3.05)	3 (0.96)	2 (0.93)	
Treatment patterns	12 (0.98)	3 (1.24)	5 (1.09)	4 (1.27)	0	
Comparative effectiveness	12 (0.98)	2 (0.83)	8 (1.74)	2 (0.64)	0	
Others	10 (0.81)	1 (0.41)	6 (1.31)	0	3 (1.4)	
Meta-analysis	5 (0.41)	3 (1.24)	1 (0.22)	1 (0.32)	0	
Disease epidemiology	7 (0.57)	3 (1.24)	2 (0.44)	0	2 (0.93)	
Literature review	6 (0.49)	1 (0.41)	2 (0.44)	0	3 (1.4)	
Systematic review	1 (0.08)	0	0	1 (0.32)	0	

Abbreviations: ASCO, American Society of Clinical Oncology; PROs, Patient-reported outcomes; RWE, real-world evidence; SGO, Society of Gynecologic Oncology.

Fisher’s exact test was applied to assess the association between the distribution of RWE abstracts based on the conference, type of analysis, and cancer type.

#### Outcomes assessed.

The most common outcome(s) assessed in RWE abstracts were molecular analyses (17.71%), followed by survival/efficacy (12.98%) and molecular analyses/survival (6.72%). About 6.32% and 3.36% of abstracts focused on screening and economic outcomes, respectively (S4 Table). Among the various cancer types analyzed, molecular analyses were the most frequently evaluated outcomes in multiple cancer abstracts (30.91%). RWE abstracts on specific gynecological cancers analyzed the survival/efficacy outcome more compared with those on multiple cancers (cervical vs ovarian vs endometrial vs multiple: 13.54% vs 15.36% vs 16.67% vs 5.85%). Interestingly, screening (14.77%) and the survival outcomes following a surgical intervention (survival/surgery; 9.85%) were commonly assessed within cervical cancer abstracts specifically (*p* < 0.001 for all comparisons) (S4 Table).

Regardless of the outcomes assessed, the majority of RWE abstracts were accepted as poster sessions. Molecular analyses (n = 311) were more likely to be accepted as poster sessions (n = 213; 68.49%) followed by publication only/abstract (n = 81; 26.05%), and oral presentations (11; 3.54%). Similarly, the majority of the survival/efficacy analyses (n = 228) were accepted as poster sessions (n = 176; 77.19%) followed by publication only/abstract (18.42% [n = 42],) and oral presentation (n = 5 [2.19%]). A higher proportion of abstracts assessing economic (20.34% vs 11.86%), healthcare resource utilization (14.29% vs 0%), efficacy and safety (12.5% vs 6.25%), and perioperative (13.33% vs 0%) outcomes were accepted as orals than publication only/abstract (*p* < 0.001 for all) (S5 Table).

## Discussion

RWE signifies a bridge between the evidence generated in CT programs and routine clinical practice. To the best of our knowledge, this study is the first to document the distribution, trends, and characteristics related to the tier of acceptance of RWE in gynecologic cancers presented at ASCO and SGO. The results showed an increase in the number of overall abstracts presented from 2018 to 2020; RWE abstracts represented a greater proportion of accepted abstracts than CTs, with an increasing trend in their acceptance during this duration. Furthermore, there were notable differences in the acceptance of abstracts between the two conferences. RWE abstracts represented a statistically higher proportion of work at SGO than at ASCO (69.99% vs 30.01%). These differences may be attributed to the distinct focus criteria of the conferences. SGO concentrates on gynecologic cancer care and emphasizes the latest scientific advancements and clinical practices specific to this field [14–16]. This aligns with higher acceptance of RWEs, providing valuable insights into real-world clinical outcomes and practices. Moreover, RWE provides a comprehensive view of treatment performance in routine clinical practice, allowing for tailored treatments to individual needs, ultimately improving outcomes and guiding policy decisions [17]. In contrast, ASCO is the largest multidisciplinary oncology conference focusing on the latest advances in clinical cancer research, particularly therapeutics [18–20]. Emphasis on CTs at ASCO highlights its role in presenting innovative clinical research and novel therapeutic approaches across various cancers. The increased acceptance of oncology RWE observed in our study is in line with previous research that reported a higher number of RWE studies between 2015 and 2019 [2]. The challenges of conducting CTs during the COVID-19 pandemic [21], coupled with the transition to virtual conferences might contribute to the higher acceptance of RWE abstracts during the study duration. This shift reflects the research community’s adaptability and the growing reliance on RWE to address emerging healthcare challenges during the pandemic. The rise in RWE abstracts has significant implications, specifically for oncology research. By providing real-world insights into the effectiveness and safety of cancer treatments, treatment patterns, patient outcomes, and health economics, RWE can inform the development of evidence-based treatment guidelines, healthcare decision-making, regulatory decisions [2,22], and reimbursement decisions [23].

Overall and among RWE specifically, ovarian cancer abstracts were the most accepted compared with other cancer types. These results may be attributable to the higher incidence of ovarian cancer population, and/or the latest breakthrough treatment with poly (ADP-ribose) polymerase inhibitors that is rapidly changing the treatment paradigms in ovarian cancer [24]. However, among CTs, most of the abstracts included were of multiple cancers. The majority of the RWE abstracts were from single sites, had academic authorship, and the data source was from North America. Furthermore, utilization of other clinical data sources, studies with statistical significance, retrospective study design, and analysis of molecular and/or survival-based outcomes were also most prevalent within RWE abstracts. Molecular outcomes were commonly reported by multiple cancer abstracts. Interestingly, screening outcomes were more often assessed in cervical cancer abstracts compared to all other cancer types.

Gynecologic cancers represent a significant cause of death worldwide and place a substantial economic and humanistic burden on women and their families [25–28]. Cervical cancer is the second most common gynecologic cancer in the majority of regions, specifically in developing countries, whereas ovarian and uterine cancers occur less frequently [25]. However, ovarian cancer has the worst prognosis and highest mortality rate among all gynecological cancers [29]. High incidence rates of ovarian cancer in the North American and West European regions may be linked to the high prevalence of several established risk factors [25]. In our study, the majority of the RWE abstracts were from North America, particularly on ovarian cancer. Also, the preponderance of retrospective RWEs noted may be due to their economical and time-efficient nature, rapid answerability to queries on adherence, and unanticipated safety concerns [30,31]. However, considering inherent limitations and the possibility of confounding and biases, data from retrospective real-world studies should be interpreted with care and a critical evaluation of their methods [17,30]. Methodological recommendations of the International Society for Pharmacoeconomics and Outcomes Research task force can be used to mitigate the biases and validity threats and to improve the causal inferences from such studies [32,33].

Furthermore, advances in molecular and genetic profiles of cancers have led to increased utilization of targeted therapies for personalized cancer care [34]. Understanding cancer genetics and the identification of predictive biomarkers are necessary to ensure better health for women with gynecologic cancer [35]. In our analysis, molecular outcomes were most commonly assessed in the RWE abstracts, thus demonstrating the increasing importance of this endpoint in research involving the management of gynecologic cancers. Also, RWE studies are useful in providing data on survival outcomes mainly *via* cancer registries in a shorter duration [1]. In our analysis, survival-based outcomes were the second most assessed, and this could be due to the rapid generation of survival data through various RWD sources. Cervical cancer is considered to be avoidable because of the effective preemptive measures including human papillomavirus vaccination and timely screening and treatment of precancerous lesions [36]. The preponderance of screening outcomes in cervical cancer abstracts noted in this study was not surprising as the guidelines for the management of cervical cancer are available in many countries [37,38].

Posters are an effective method for knowledge transfer at oncology conferences. Significant advantages of posters include availability for a longer duration, discussions, and networking opportunities with the authors leading to collaborations [39,40]. Our study results show that both RWE and CT abstracts were more likely to be accepted as posters, though oral presentations had a major presence among CTs. The higher acceptance of RWE abstracts as posters in this study indicates that they do not always achieve the visibility of CTs. A higher proportion of RWE abstracts were accepted as publication only/abstracts at ASCO (60.34%), while as poster sessions at SGO (91.62%).

Azad et al, reported a statistically significant association between the involvement of multiple institutions and larger population size with the RWE acceptance category for publication only versus oral/poster presentations [41]. In our study, RWE abstracts were more commonly accepted as poster sessions regardless of the various types of data collected, statistical significance, study design, and the outcomes assessed. Statistical analysis is a crucial part of any clinical and translational research [42]. In the present study, nearly one-third of RWE abstracts (31.49%) did not report a statistical analysis of the results, demonstrating a piece of suboptimal information. Reporting statistical analysis in the RWE abstracts can help the readers to determine the robustness of study data and can improve the visibility in the scientific community. To enhance statistical reporting in RWE, it is recommended that conferences include this criterion in the abstract submission guidelines mandating key statistical metrics. These measures may aid in quality improvement, higher-tier acceptance, and wider dissemination of novel RWE findings among the oncology scientific community.

### Strengths and limitations

The major strength of this study is that it reinforces the increasing distribution of RWEs in cancer care. This is the first study reporting multiple characteristics of RWE abstracts on gynecological cancers that can influence their tier of acceptance at scientific conferences, thus providing a valuable framework for the dissemination of clinical oncology research. Additionally, the analysis performed for studies accepted before and during the COVID-19 pandemic provides details on how COVID-19 might have influenced the acceptance rate of both CT and RWE abstracts at conferences.

However, there are important limitations associated with this study. This study did not evaluate the data from the European Society for Medical Oncology (ESMO) congress and included only the conferences conducted in the US. A comparison with ESMO may have indicated a European perspective of RWE data. Predisposed publication and submission biases have to be accounted for while interpreting the results of the present study. Submission biases related to geographic regions or funding sources can affect the type of studies submitted and accepted at these conferences. Research from more economically developed regions or well-funded institutions may be favored [43], potentially influencing the study findings. The fact that both conferences were held in the US and two thirds of the data sources for the abstracts were from North America could reflect geographical bias. This may limit the generalizability of the study’s conclusions and not fully capture the global landscape of research. Additionally, in the absence of access to the rejected abstracts, the present study categorization of abstracts may not reflect all submissions.

Moreover, a three-year timeframe can be too short to fully establish any trends. The virtual format of conferences in 2020 may have changed the number of submissions and influenced the acceptance types to accommodate this new setup. As the study was limited to evaluating the abstracts *via* the information included in them, which is subject to strict word count and other limits, there could be important details in the study that may be missed. For approximately 80% of the RWE, the industry funding details were not available in the published abstracts. Due to the retrospective nature of the present study, it was not feasible to collect these funding details, which may be otherwise included in the actual oral or poster presentations at the conferences. While it is possible that funding details were disclosed to the conference, they may not have been accessible in the abstract data available to us. Additionally, the absence of funding information in RWE abstracts could impact the acceptance at conferences, as industry funding often directs research priorities, introduces potential reporting bias, and influences the perceived credibility and ethical considerations of the research [44]. Also, some studies did not provide details of the study design, statistical significance of results and *p*-values, and sample size. Since only the abstract was accessible, quality was not assessed. Another limitation of the study while comparing the conferences is that the hierarchal orders of acceptances at each conference vary, and they are not necessarily equivalent to one another. Finally, this study was limited to evaluating the gynecologic cancer abstracts. Future research assessing the abstract presentation patterns of other cancers, specifically more highly funded types such as breast, hematological, and brain [45], may reveal evolving research trends and identify gaps in the dissemination of novel research for these cancer types as well.

## Conclusions

RWE abstract data were more frequently presented than CTs at SGO versus ASCO, indicating their substantial contribution to the gynecologic oncology scientific community. Additionally, RWEs were more likely to be accepted as posters, with a smaller proportion of RWE abstracts accepted as oral presentations versus CTs across conferences and cancer types. RWE abstracts utilizing other clinical data sources, analyzing molecular and/or survival outcomes, and reporting statistically significant results were the most common. The results demonstrate that while RWE studies are prevalent, they may not always achieve the visibility of CTs in providing valuable data for healthcare decision-making. The study findings may guide researchers in understanding the type of abstracts preferred at conferences and how best to disseminate their study results to the oncology community.

## Supporting information

S1 Fig Distribution of RWE studies stratified by (A) Study site, (B) Academic authorship (yes, no, or both refer to studies including academic authorship, no academic authorship, or both academic authorship and research funding), and (C) Region of data source. Abbreviations: RWE, real-world evidence.
(DOCX)

S1 Table Search strategy to identify CT and RWE abstracts. Abbreviations: CT, clinical trial; EHR, electronic health record; EMR, electronic medical record; PRO, patient-reported outcome; RWD, real word data; RWE, real-world evidence. Published PDFs of the ASCO and SGO annual meeting proceedings were referred to for identifying the abstracts if available.
(DOCX)

S2 Table Distribution of RWE abstracts by type of data collected and tier of acceptance. Abbreviations: EHR, electronic health record; EMR, electronic medical record; RWE, real-world evidence. Fisher’s exact test was applied to assess the association between the distribution of RWE abstracts based on the type of data collected and tier of acceptance. Of the RWE abstracts (N = 1756) there were a total of 69 orals, 10 poster discussion sessions, 1315 poster sessions, 15 international sessions, 10 education forums, 328 publication only/abstract, and 9 others. ^a^Others category included abstracts that could not be classified into the various categories of “Type of data collected”. ^b^Special interest session and Sunrise seminar were combined into the “Others category”.
(DOCX)

S3 Table Distribution of RWE abstracts by type of analysis and tier of acceptance. Abbreviations: PROs, Patient-reported outcomes; RWE, real-world evidence. Fisher’s exact test was applied to assess the association between the distribution of RWE abstracts based on the type of analysis and tier of acceptance. Of the RWE abstracts (N = 1756) there were a total of 69 orals, 10 poster discussion sessions, 1315 poster sessions, 15 international sessions, 10 education forums, 328 publication only/abstract, and 9 others. ^a^Special interest session and Sunrise seminar were combined into the “Others category”.
(DOCX)

S4 Table Distribution of the RWE studies by outcomes assessed and cancer type. Abbreviations: HCRU, healthcare resource utilization; HRQoL, health-related quality of life; RWE, real-world evidence. Fisher’s exact test was applied to assess the association between the distribution of RWE abstracts based on the outcomes assessed and cancer type. The categorization for the outcome assessed was based on the key outcomes reported in each abstract. ^a^Others categories included abstracts that could not be classified into the above-mentioned categories of outcomes assessed.
(DOCX)

S5 Table Distribution of RWE abstracts by outcomes assessed and tier of acceptance. Abbreviations: HCRU, healthcare resource utilization; HRQoL, health-related quality of life; RWE, real-world evidence. Fisher’s exact test was applied to assess the association between the distribution of RWE abstracts based on the outcomes assessed and tier of acceptance. The categorization for the outcomes assessed was based on the key outcomes reported in each abstract. ^a^Others category included abstracts that could not be classified into the above-mentioned categories of outcomes assessed. ^b^Special interest session and Sunrise seminar were combined into the “Others category”.
(DOCX)
