# Supplementary material for: Real-world evidence in gynecologic cancers presented at key oncology conferences in the United States: Distribution and factors related to high-tier acceptance
Source: PLoS One. 2025 Apr 22;20(4):e0321654. doi: 10.1371/journal.pone.0321654 (PMC12013925; doi:10.1371/journal.pone.0321654)
Supplement: S1 Fig — Abbreviations: RWE, real-world evidence. (DOCX) [file pone.0321654.s001.docx]

**S1 Fig. Distribution of RWE studies stratified by (A) Study site, (B) Academic authorship (yes, no, or both: refer to studies including academic authorship, no academic authorship, or both academic authorship and research funding), and (C) Region of data source.**

**(A) Study site**


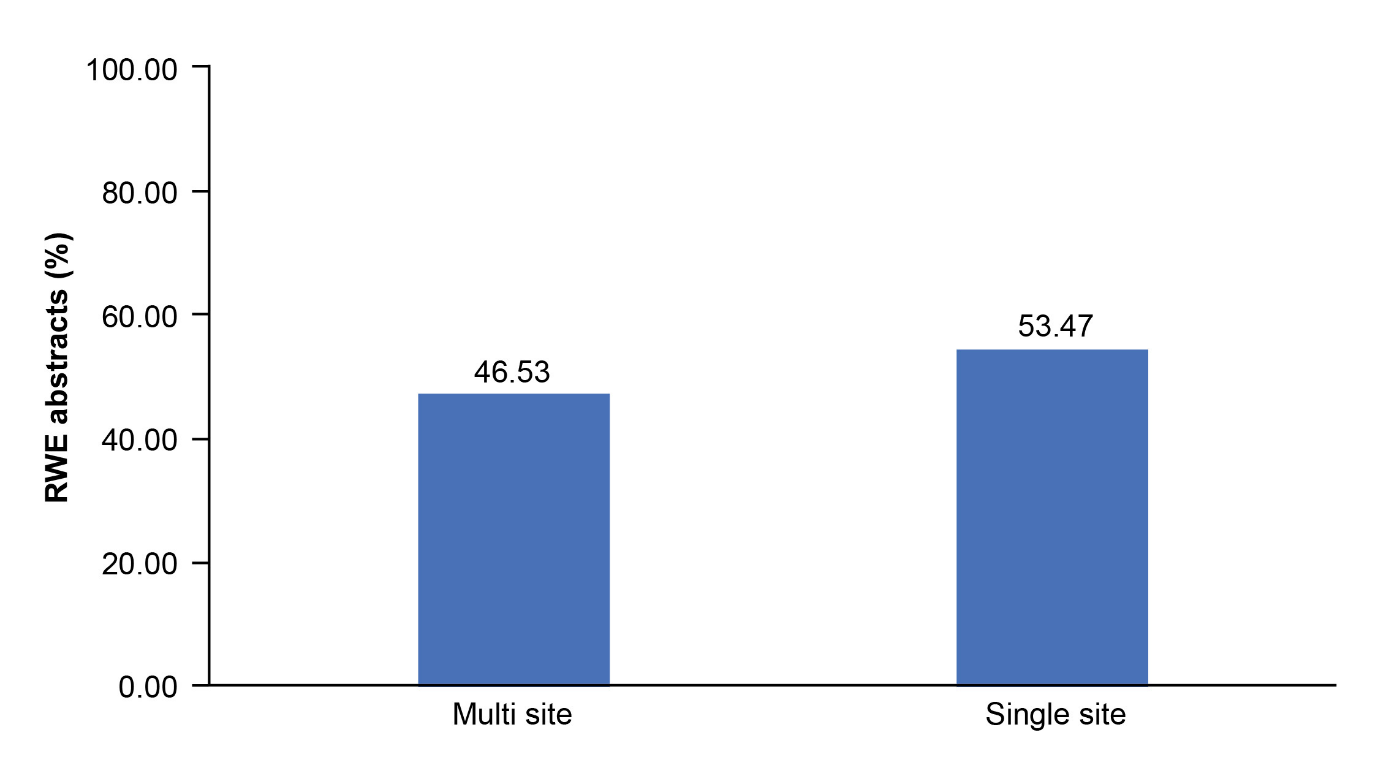


**(B) Academic authorship (yes, no, or both: refer to studies including academic authorship, no academic authorship, or both academic authorship and research funding)**


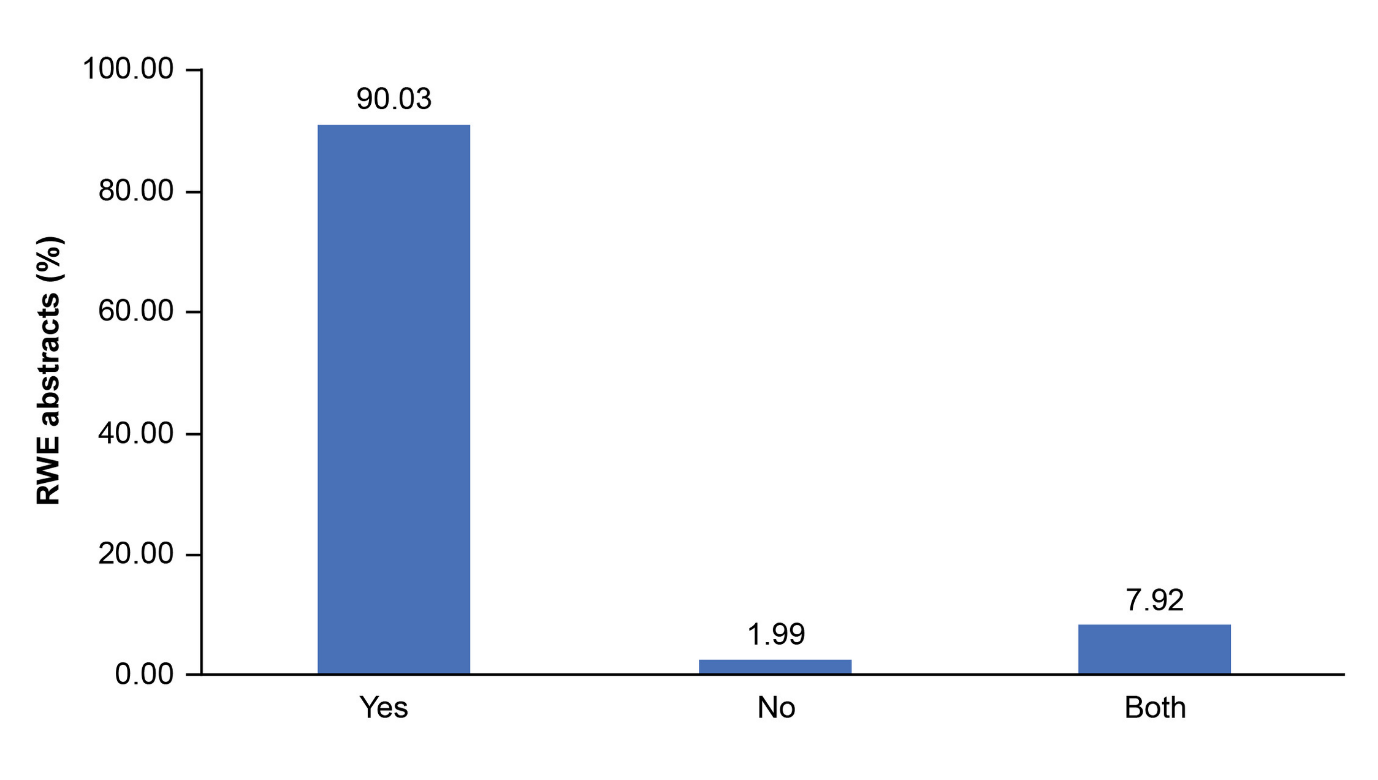


**(C) Region of data source**


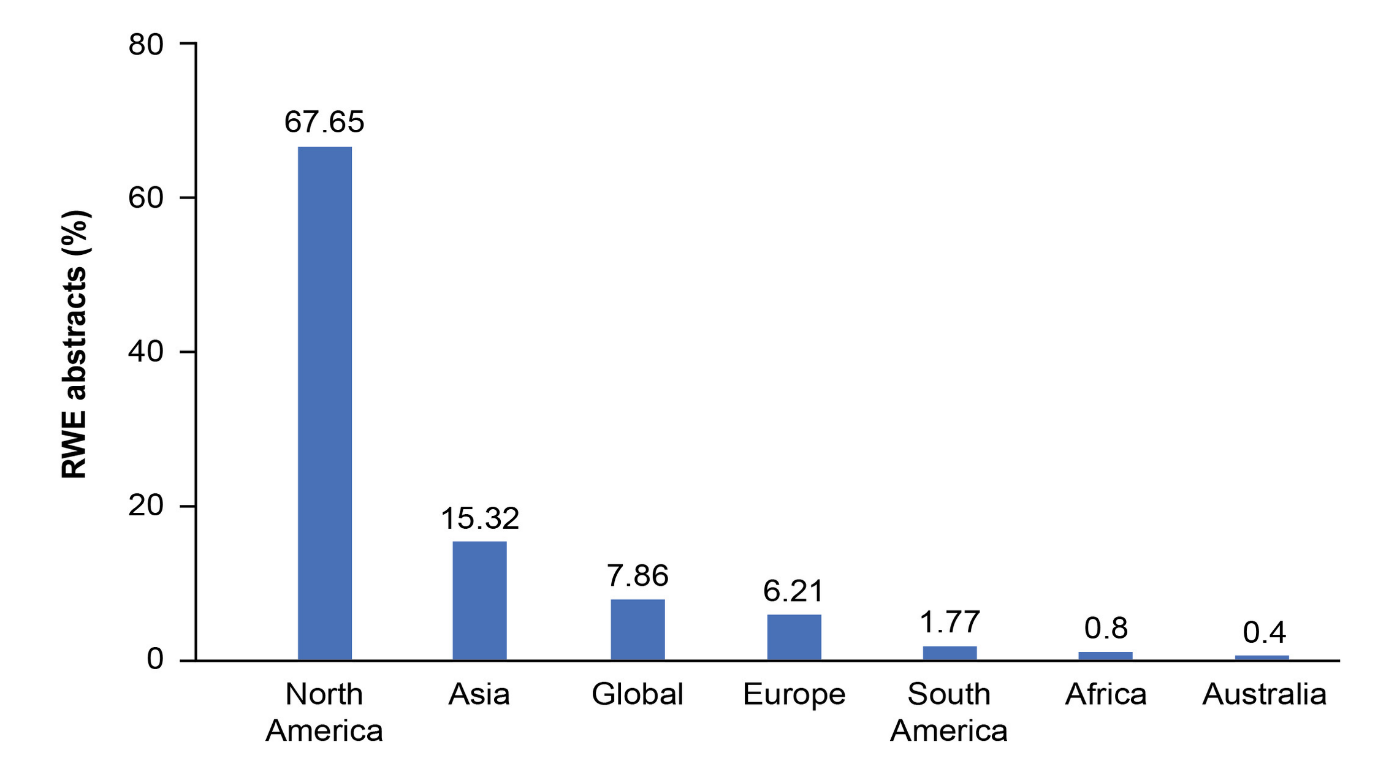


Abbreviations: RWE, real-world evidence.
