# Supplementary material for: Real-world evidence in gynecologic cancers presented at key oncology conferences in the United States: Distribution and factors related to high-tier acceptance
Source: PLoS One. 2025 Apr 22;20(4):e0321654. doi: 10.1371/journal.pone.0321654 (PMC12013925; doi:10.1371/journal.pone.0321654)
Supplement: S1 Table — Abbreviations: CT, clinical trial; EHR, electronic health record; EMR, electronic medical record; PRO, patient-reported outcome; RWD, real word data; RWE, real-world evidence. Published PDFs of the ASCO and SGO annual meeting proceedings were referred to for identifying the abstracts if available. (DOCX) [file pone.0321654.s002.docx]

**S1 Table. Search strategy to identify CT and RWE abstracts.**

| **Embase** | |
| --- | --- |
| #1 | 'cervical cancer'/exp OR 'cervical cancer' OR 'cervical tumor'/exp OR 'cervical tumor' OR 'cervical carcinoma'/exp OR 'cervical carcinoma' |
| #2 | 'ovarian cancer'/exp OR 'ovarian cancer' OR 'ovarian tumor'/exp OR 'ovarian tumor' OR 'ovarian carcinoma'/exp OR 'ovarian carcinoma' |
| #3 | 'endometrial cancer'/exp OR 'endometrial cancer' OR 'endometrial tumor'/exp OR 'endometrial tumor' OR 'endometrial carcinoma'/exp OR 'endometrial carcinoma' |
| #4 | #1 OR #2 OR #3 |
| #5 | #4 AND ([conference abstract]/lim OR [conference paper]/lim OR [conference review]/lim) AND [2018-2021]/py |
| #6 | #5 AND ('2018 annual meeting of the american society of clinical oncology, asco 2018':nc OR '2019 annual meeting of the american society of clinical oncology, asco 2019':nc OR '2020 annual meeting of the american society of clinical oncology, asco 2020':nc) |
| #7 | #5 AND ('50th annual meeting of the society of gynecologic oncology':nc OR 'sgo 2020 annual meeting':nc OR 'society of gynecologic oncology 2020 annual meeting on women`s cancer':nc) |
| **SGO and ASCO (2018 – 2020)** | |
| #1 | Terms used for manual search: 'cervical cancer', 'cervical tumor', 'cervical carcinoma', 'ovarian cancer', 'ovarian tumor', 'ovarian carcinoma', 'endometrial cancer', 'endometrial tumor', 'endometrial carcinoma' |
| **Additional terms considered to identify CT and RWE abstracts** | |
| CT: clinical trial, randomized controlled trial, phase 1 trial, phase 2 trial, phase 3 trial, phase, placebo, clinical study | |
| RWE: real world/real-world, observational, non-interventional, non-interventional, retrospective, cohort, survey, cross sectional/cross-sectional, case-control/case control, database, electronic health record, EHR, RWE, RWD, electronic medical record, EMR, claims, registry, PRO, patient-reported outcome/patient reported outcome, chart review, healthcare resource use, utilization, treatment pattern, interviews, burden of illness, burden of disease, economic burden, humanistic burden, adherence, comparative effectiveness, case series, prospective | |

Abbreviations: CT, clinical trial; EHR, electronic health record; EMR, electronic medical record; PRO, patient-reported outcome; RWD, real word data; RWE, real-world evidence.

Published PDFs of the ASCO and SGO annual meeting proceedings were referred to for identifying the abstracts if available.
