# Supplementary material for: Real-world evidence in gynecologic cancers presented at key oncology conferences in the United States: Distribution and factors related to high-tier acceptance
Source: PLoS One. 2025 Apr 22;20(4):e0321654. doi: 10.1371/journal.pone.0321654 (PMC12013925; doi:10.1371/journal.pone.0321654)
Supplement: S2 Table — Abbreviations: EHR, electronic health record; EMR, electronic medical record; RWE, real-world evidence. Fisher’s exact test was applied to assess the association between the distribution of RWE abstracts based on the type of data collected and tier of acceptance. Of the RWE abstracts (N = 1756) there were a total of 69 orals, 10 poster discussion sessions, 1315 poster sessions, 15 international sessions, 10 education forums, 328 publication only/abstract, and 9 others. aOthers category included abstracts that could not be classified into the various categories of “Type of data collected”. bSpecial interest session and Sunrise seminar were combined into the “Others category”. (DOCX) [file pone.0321654.s003.docx]

**S2 Table. Distribution of RWE abstracts by type of data collected and tier of acceptance.**

| **Type of data collected** | **Total**  **(N = 1756)**  **n (%)** | **Oral**  **n (%)** | **Poster Discussion Session**  **n (%)** | **Poster Session**  **n (%)** | **International Session**  **n (%)** | **Education Forum**  **n (%)** | **Publication only/Abstract**  **n (%)** | **Others^b^**  **n (%)** | ***p*-value** |
| --- | --- | --- | --- | --- | --- | --- | --- | --- | --- |
| Other clinical data sources | 783 (44.59) | 23 (2.94) | 7 (0.89) | 584 (74.58) | 10 (1.28) | 4 (0.51) | 149 (19.03) | 6 (0.77) | 0.2509 |
| Other data repository | 378 (21.53) | 20 (5.29) | 1 (0.26) | 282 (74.6) | 2 (0.53) | 1 (0.26) | 72 (19.05) | 0 |  |
| Medical records | 117 (6.66) | 5 (4.27) | 0 | 95 (81.2) | 2 (1.71) | 0 | 15 (12.82) | 0 |  |
| Chart review | 105 (5.98) | 3 (2.86) | 1 (0.95) | 83 (79.05) | 0 | 0 | 18 (17.14) | 0 |  |
| Other primary data collection | 88 (5.01) | 5 (5.68) | 1 (1.14) | 59 (67.05) | 0 | 2 (2.27) | 20 (22.73) | 1 (1.14) |  |
| Survey | 82 (4.67) | 4 (4.88) | 0 | 64 (78.05) | 0 | 0 | 12 (14.63) | 2 (2.44) |  |
| Patient registry | 53 (3.02) | 1 (1.89) | 0 | 40 (75.47) | 0 | 1 (1.89) | 11 (20.75) | 0 |  |
| Others^a^ | 47 (2.68) | 1 (2.13) | 0 | 36 (76.6) | 0 | 0 | 10 (21.28) | 0 |  |
| EMR | 31 (1.77) | 2 (6.45) | 0 | 27 (87.1) | 0 | 0 | 2 (6.45) | 0 |  |
| Claims data | 20 (1.14) | 2 (10) | 0 | 11 (55) | 0 | 2 (10) | 5 (25) | 0 |  |
| Interview | 17 (0.97) | 2 (11.76) | 0 | 9 (52.94) | 1 (5.88) | 0 | 5 (29.41) | 0 |  |
| Literature | 9 (0.51) | 0 | 0 | 7 (77.78) | 0 | 0 | 2 (22.22) | 0 |  |
| Medical records and patient registry | 9 (0.51) | 0 | 0 | 8 (88.89) | 0 | 0 | 1 (11.11) | 0 |  |
| Billing data | 6 (0.34) | 0 | 0 | 4 (66.67) | 0 | 0 | 2 (33.33) | 0 |  |
| Questionnaire | 6 (0.34) | 1 (16.67) | 0 | 4 (66.67) | 0 | 0 | 1 (16.67) | 0 |  |
| EHR | 5 (0.28) | 0 | 0 | 2 (40) | 0 | 0 | 3 (60) | 0 |  |

Abbreviations: EHR, electronic health record; EMR, electronic medical record; RWE, real-world evidence.

Fisher's exact test was applied to assess the association between the distribution of RWE abstracts based on the type of data collected and tier of acceptance.

Of the RWE abstracts (N = 1756) there were a total of 69 orals, 10 poster discussion sessions, 1315 poster sessions, 15 international sessions, 10 education forums, 328 publication only/abstract, and 9 others.

^a^Others category included abstracts that could not be classified into the various categories of “Type of data collected”.

^b^Special interest session and Sunrise seminar were combined into the “Others category”.
