# Supplementary material for: Real-world evidence in gynecologic cancers presented at key oncology conferences in the United States: Distribution and factors related to high-tier acceptance
Source: PLoS One. 2025 Apr 22;20(4):e0321654. doi: 10.1371/journal.pone.0321654 (PMC12013925; doi:10.1371/journal.pone.0321654)
Supplement: S3 Table — Abbreviations: PROs, Patient-reported outcomes; RWE, real-world evidence. Fisher’s exact test was applied to assess the association between the distribution of RWE abstracts based on the type of analysis and tier of acceptance. Of the RWE abstracts (N = 1756) there were a total of 69 orals, 10 poster discussion sessions, 1315 poster sessions, 15 international sessions, 10 education forums, 328 publication only/abstract, and 9 others. aSpecial interest session and Sunrise seminar were combined into the “Others category”. (DOCX) [file pone.0321654.s004.docx]

**S3 Table. Distribution of RWE abstracts by type of analysis and tier of acceptance.**

| **Type of analysis** | **Total (N = 1756)**  **n (%)** | **Oral**  **n (%)** | **Poster Discussion Session**  **n (%)** | **Poster Session**  **n (%)** | **International Session**  **n (%)** | **Education Forum**  **n (%)** | **Publication only/Abstract**  **n (%)** | **Others^a^**  **n (%)** | ***p*-value** |
| --- | --- | --- | --- | --- | --- | --- | --- | --- | --- |
| Retrospective | 1053 (59.97) | 33 (3.13) | 4 (0.38) | 824 (78.25) | 8 (0.76) | 6 (0.57) | 174 (16.52) | 4 (0.38) | < 0.005 |
| Diagnosis | 281 (16) | 9 (3.2) | 4 (1.42) | 197 (70.11) | 4 (1.42) | 0 | 65 (23.13) | 2 (0.71) |  |
| Prospective | 165 (9.4) | 11 (6.67) | 1 (0.61) | 118 (71.52) | 2 (1.21) | 0 | 33 (20) | 0 |  |
| PROs | 72 (4.1) | 5 (6.94) | 1 (1.39) | 49 (68.06) | 0 | 0 | 15 (20.83) | 2 (2.78) |  |
| Survival | 57 (3.25) | 2 (3.51) | 0 | 46 (80.7) | 0 | 0 | 9 (15.79) | 0 |  |
| Economic model | 29 (1.65) | 5 (17.24) | 0 | 17 (58.62) | 1 (3.45) | 3 (10.34) | 3 (10.34) | 0 |  |
| Comparative effectiveness | 22 (1.25) | 1 (4.55) | 0 | 15 (68.18) | 0 | 1 (4.55) | 5 (22.73) | 0 |  |
| Treatment patterns | 21 (1.2) | 1 (4.76) | 0 | 14 (66.67) | 0 | 0 | 6 (28.57) | 0 |  |
| Literature review | 12 (0.68) | 1 (8.33) | 0 | 6 (50) | 0 | 0 | 4 (33.33) | 1 (8.33) |  |
| Meta-analysis | 17 (0.97) | 0 | 0 | 9 (52.94) | 0 | 0 | 8 (47.06) | 0 |  |
| Others | 14 (0.8) | 1 (7.14) | 0 | 11 (78.57) | 0 | 0 | 2 (14.29) | 0 |  |
| Disease epidemiology | 10 (0.57) | 0 | 0 | 8 (0.61) | 0 | 0 | 2 (0.61) | 0 |  |
| Systematic review | 3 (0.17) | 0 | 0 | 1 (33.33) | 0 | 0 | 2 (66.67) | 0 |  |

Abbreviations: PROs, Patient-reported outcomes; RWE, real-world evidence.

Fisher's exact test was applied to assess the association between the distribution of RWE abstracts based on the type of analysis and tier of acceptance.

Of the RWE abstracts (N = 1756) there were a total of 69 orals, 10 poster discussion sessions, 1315 poster sessions, 15 international sessions, 10 education forums, 328 publication only/abstract, and 9 others.

^a^Special interest session and Sunrise seminar were combined into the “Others category”.
