# Supplementary material for: Real-world evidence in gynecologic cancers presented at key oncology conferences in the United States: Distribution and factors related to high-tier acceptance
Source: PLoS One. 2025 Apr 22;20(4):e0321654. doi: 10.1371/journal.pone.0321654 (PMC12013925; doi:10.1371/journal.pone.0321654)
Supplement: S4 Table — Abbreviations: HCRU, healthcare resource utilization; HRQoL, health-related quality of life; RWE, real-world evidence. Fisher’s exact test was applied to assess the association between the distribution of RWE abstracts based on the outcomes assessed and cancer type. The categorization for the outcome assessed was based on the key outcomes reported in each abstract. aOthers categories included abstracts that could not be classified into the above-mentioned categories of outcomes assessed. (DOCX) [file pone.0321654.s005.docx]

**S4 Table. Distribution of the RWE studies by outcomes assessed and cancer type.**

| **Outcomes assessed** | **Total studies**  **(N = 1756)**  **N (%)** | **Cervical cancer**  **(n = 325)**  **n (%)** | **Ovarian cancer**  **(n = 638)**  **n (%)** | **Endometrial cancer**  **(n = 366)**  **n (%)** | **Multiple cancers**  **(n = 427)**  **n (%)** | ***p*-value** |
| --- | --- | --- | --- | --- | --- | --- |
| Molecular analyses | 311 (17.71) | 16 (4.92) | 108 (16.93) | 55 (15.03) | 132 (30.91) | < 0.001 |
| Survival/Efficacy | 228 (12.98) | 44 (13.54) | 98 (15.36) | 61 (16.67) | 25 (5.85) |  |
| Molecular analyses/Survival | 118 (6.72) | 12 (3.69) | 58 (9.09) | 32 (8.74) | 16 (3.75) |  |
| Screening | 111 (6.32) | 48 (14.77) | 16 (2.51) | 21 (5.74) | 26 (6.09) |  |
| Patient characteristics | 98 (5.58) | 17 (5.23) | 22 (3.45) | 26 (7.1) | 33 (7.73) |  |
| Survival/Surgery | 98 (5.58) | 32 (9.85) | 39 (6.11) | 19 (5.19) | 8 (1.87) |  |
| Patient characteristics/Survival | 93 (5.3) | 13 (4.0) | 44 (6.9) | 21 (5.74) | 15 (3.51) |  |
| HRQoL | 92 (5.24) | 10 (3.08) | 28 (4.39) | 17 (4.64) | 37 (8.67) |  |
| Postoperative outcomes | 71 (4.04) | 11 (3.38) | 31 (4.86) | 15 (4.1) | 14 (3.28) |  |
| Economic | 59 (3.36) | 12 (3.69) | 29 (4.55) | 6 (1.64) | 12 (2.81) |  |
| Survival/Efficacy and safety | 53 (3.02) | 14 (4.31) | 28 (4.39) | 4 (1.09) | 7 (1.64) |  |
| Surgical outcomes | 45 (2.56) | 12 (3.69) | 23 (3.61) | 8 (2.19) | 2 (0.47) |  |
| Tumor characteristics | 43 (2.45) | 12 (3.69) | 13 (2.04) | 14 (3.83) | 4 (0.94) |  |
| Safety | 42 (2.39) | 6 (1.85) | 16 (2.51) | 2 (0.55) | 18 (4.22) |  |
| Others^a^ | 40 (2.28) | 9 (2.77) | 13 (2.04) | 5 (1.37) | 13 (3.04) |  |
| Survival | 39 (2.22) | 4 (1.23) | 7 (1.1) | 21 (5.74) | 7 (1.64) |  |
| Tumor characteristics/Survival | 36 (2.05) | 13 (4.0) | 8 (1.25) | 8 (2.19) | 7 (1.64) |  |
| Incidence/Prevalence | 31 (1.77) | 8 (2.46) | 5 (0.78) | 7 (1.91) | 11 (2.58) |  |
| Efficacy | 25 (1.42) | 3 (0.92) | 8 (1.25) | 8 (2.19) | 6 (1.41) |  |
| Treatment decision-making | 25 (1.42) | 3 (0.92) | 10 (1.57) | 3 (0.82) | 9 (2.11) |  |
| HCRU | 21 (1.2) | 2 (0.62) | 6 (0.94) | 1 (0.27) | 12 (2.81) |  |
| Efficacy and safety | 16 (0.91) | 2 (0.62) | 8 (1.25) | 4 (1.09) | 2 (0.47) |  |
| Perioperative outcomes | 15 (0.85) | 6 (1.85) | 7 (1.1) | 1 (0.27) | 1 (0.23) |  |
| Treatment guideline adherence | 14 (0.8) | 6 (1.85) | 4 (0.63) | 1 (0.27) | 3 (0.7) |  |
| Practice patterns | 13 (0.74) | 4 (1.23) | 4 (0.63) | 1 (0.27) | 4 (0.94) |  |
| Survival/Safety | 9 (0.51) | 1 (0.31) | 2 (0.31) | 5 (1.37) | 1 (0.23) |  |
| Preoperative outcomes | 5 (0.28) | 2 (0.62) | 3 (0.47) | 0 | 0 |  |
| Safety/Surgery | 5 (0.28) | 3 (0.92) | 0 | 0 | 2 (0.47) |  |

Abbreviations: HCRU, healthcare resource utilization; HRQoL, health-related quality of life; RWE, real-world evidence.

Fisher's exact test was applied to assess the association between the distribution of RWE abstracts based on the outcomes assessed and cancer type.

The categorization for the outcome assessed was based on the key outcomes reported in each abstract.

^a^Others categories included abstracts that could not be classified into the above-mentioned categories of outcomes assessed.
